# The Influence of Pubertal Development on Autoantibody Appearance and Progression to Type 1 Diabetes in the TEDDY Study

**DOI:** 10.1210/jendso/bvae103

**Published:** 2024-05-24

**Authors:** Katharina Warncke, Roy Tamura, Desmond A Schatz, Riitta Veijola, Andrea K Steck, Beena Akolkar, William Hagopian, Jeffrey P Krischer, Åke Lernmark, Marian J Rewers, Jorma Toppari, Richard McIndoe, Anette-G Ziegler, Kendra Vehik, Michael J Haller, Helena Elding Larsson

**Affiliations:** TUM School of Medicine, Department of Pediatrics, Technical University of Munich, 81675 Munich, Germany; German Center for Environmental Health, Institute of Diabetes Research, Helmholtz Munich, 80939 Munich, Germany; Health Informatics Institute, University of South Florida, Tampa, FL 33612, USA; Diabetes Center of Excellence, University of Florida, Gainesville, FL 32610, USA; Department of Pediatrics, Research Unit of Clinical Medicine, Medical Research Center Oulu, University of Oulu and Oulu University Hospital, 90014 Oulu, Finland; Barbara Davis Center for Diabetes, University of Colorado Anschutz Medical Campus, Aurora, CO 80045, USA; National Institute of Diabetes and Digestive and Kidney Diseases, Bethesda, MD 20892, USA; Department of Pediatrics, Indiana University School of Medicine, Indianapolis, IN 46202, USA; Health Informatics Institute, University of South Florida, Tampa, FL 33612, USA; Department of Clinical Sciences, Lund University/Clinical Research Centre, Skane University Hospital, 21428 Malmö, Sweden; Barbara Davis Center for Diabetes, University of Colorado Anschutz Medical Campus, Aurora, CO 80045, USA; Department of Pediatrics, University of Turku and Turku University Hospital, 20520 Turku, Finland; Institute of Biomedicine, Research Centre for Integrative Physiology and Pharmacology, and Centre for Population Health Research, University of Turku, 20520 Turku, Finland; Center for Biotechnology and Genomic Medicine, Medical College of Georgia, Augusta University, Augusta, GA 30912, USA; German Center for Environmental Health, Institute of Diabetes Research, Helmholtz Munich, 80939 Munich, Germany; German Center for Diabetes Research (DZD), 85764 Munich-Neuherberg, Germany; Forschergruppe Diabetes, School of Medicine, Klinikum rechts der Isar, Technical University Munich, 81675 Munich, Germany; Forschergruppe Diabetes e.V. at Helmholtz Munich, German Research Center for Environmental Health, 80939 Munich, Germany; Health Informatics Institute, University of South Florida, Tampa, FL 33612, USA; Department of Pediatrics, Diabetes Institute, College of Medicine, University of Florida, Gainesville, FL 32610, USA; Unit for Pediatric Endocrinology, Department of Clinical Sciences Malmö, Lund University, 20502 Malmö, Sweden; Department of Paediatrics, Skåne University Hospital, 20502 Malmö, Sweden

**Keywords:** diabetes, β-cell, insulin resistance, type 1 diabetes

## Abstract

**Context:**

The 2 peaks of type 1 diabetes incidence occur during early childhood and puberty.

**Objective:**

We sought to better understand the relationship between puberty, islet autoimmunity, and type 1 diabetes.

**Methods:**

The relationships between puberty, islet autoimmunity, and progression to type 1 diabetes were investigated prospectively in children followed in The Environmental Determinants of Diabetes in the Young (TEDDY) study. Onset of puberty was determined by subject self-assessment of Tanner stages. Associations between speed of pubertal progression, pubertal growth, weight gain, homeostasis model assessment of insulin resistance (HOMA-IR), islet autoimmunity, and progression to type 1 diabetes were assessed. The influence of individual factors was analyzed using Cox proportional hazard ratios.

**Results:**

Out of 5677 children who were still in the study at age 8 years, 95% reported at least 1 Tanner Stage score and were included in the study. Children at puberty (Tanner Stage ≥2) had a lower risk (HR 0.65, 95% CI 0.45-0.93; *P* = .019) for incident autoimmunity than prepubertal children (Tanner Stage 1). An increase of body mass index Z-score was associated with a higher risk (HR 2.88, 95% CI 1.61-5.15; *P* < .001) of incident insulin autoantibodies. In children with multiple autoantibodies, neither HOMA-IR nor rate of progression to Tanner Stage 4 were associated with progression to type 1 diabetes.

**Conclusion:**

Rapid weight gain during puberty is associated with development of islet autoimmunity. Puberty itself had no significant influence on the appearance of autoantibodies or type 1 diabetes. Further studies are needed to better understand the underlying mechanisms.

There are 2 peaks of incidence of type 1 diabetes in childhood and adolescence, 1 in early childhood and 1 in early puberty [1-4]. While prospective studies from birth have provided ample knowledge of the natural history of type 1 diabetes during early childhood [5, 6], less is known about the incidence peak at adolescence. There are several studies that report that decreased insulin sensitivity may lead to the development of type 1 diabetes [7-9]. Indeed, insulin resistance associated with pubertal progression has been hypothesized as a potential mediator of the disease process. Data from the prospective BABYDIAB and TEENDIAB study showed a constant rise of insulin resistance in first-degree relatives from age 5 to 13 years [10]. The “accelerator hypothesis” suggests that weight gain causes insulin resistance, increases beta cell stress, and drives processes that lead to type 1 diabetes [11]. Furthermore, according to this hypothesis, rising blood glucose accelerates β-cell apoptosis (glucotoxicity), exposes additional beta-cell immunogens, and further accelerates the disease process in a subset of genetically predisposed individuals [11]. The Environmental Determinants of Diabetes in the Young (TEDDY) study prospectively examines children with an increased genetic risk of type 1 diabetes from birth through 15 years of age [12], and collects data on nutrition, diseases, environmental factors and pubertal development, among other things. In addition, regular blood samples are taken. As such, TEDDY is ideally suited to investigating the role of puberty on the initiation of autoimmunity, as well as its role in the progression of clinical type 1 diabetes. As TEDDY is nearing completing of its planned prospective follow-up, all subjects have reached puberty, making it possible to analyze the relationship between pubertal development, autoimmunity and type 1 diabetes. As such, we sought to investigate if (1) onset of puberty or (2) increased growth and weight gain during puberty affects the risk of seroconversion or progression to type 1 diabetes, and if (3) the speed of puberty development or (4) the presence of insulin resistance affects the risk of developing type 1 diabetes in children with multiple autoantibodies.

## Materials and Methods

### The TEDDY Cohort

TEDDY is a prospective cohort study funded by the National Institutes of Health with the primary goal to identify environmental causes of type 1 diabetes. It includes 6 clinical research centers—3 in the United States: Colorado, Georgia/Florida, Washington, and 3 in Europe: Finland, Germany, and Sweden. Detailed study design and methods have been previously published [13, 14]. Written informed consents were obtained for all study participants from a parent or primary caretaker, separately, for genetic screening and participation in prospective follow-up. The study was approved by local Institutional Review Boards and is monitored by External Advisory Board formed by the National Institutes of Health.

### Data

The data analyzed were based on the frozen TEDDY dataset of May 31, 2022. Out of the 5677 TEDDY subjects that were still in the study at age 8, 5379 (95%) reported at least 1 Tanner Stage score.

We utilized the TEDDY data set to explore 3 different questions: (1) Does the onset of puberty affect the risk of either seroconversion or progression to type 1 diabetes in autoantibody-positive children? (2) Once a child starts puberty, do the pubertal characteristics of increased growth and weight gain affect the risk of seroconversion or progression to type 1 diabetes? (3) For children with multiple autoantibodies, does the speed of puberty development or the presence of insulin resistance affect the risk of developing type 1 diabetes?

To answer these questions, we investigated 3 different cohorts.

Cohort 1 was defined as those subjects who were autoantibody negative at age 8 (n = 4949). Cohort 1 was utilized to investigate the development of autoantibodies and the progression to type 1 diabetes in high genetic risk children without prior evidence of autoimmunity. Using the same methodology as the original TEDDY protocol [13], cohort 1 had statistical power to detect a hazard ratio (HR) of approximately 1.8 over a 7-year follow-up.

Cohort 2 was defined as the subjects who became autoantibody positive before reaching Tanner Stage 2 (n = 430). This cohort was very well suited to investigate the effects of puberty on progression from autoimmunity to clinical type 1 diabetes.

Cohort 3 was defined as the subjects who reported a Tanner Stage greater than 1 (n = 4857). Cohort 3 was used to investigate the broader effects of puberty on development of autoimmunity and type 1 diabetes. Tables 1 and 2 show basic demographics of these 3 cohorts.

**Table 1.** Demographics for autoantibody-negative subjects (cohort 1; n = 4949) and autoantibody positive subjects at age 8 (cohort 2; n = 430)

Variable	Cohort 1; n = 4949	Cohort 2; n = 430
Site		
United States, n (%)	1955 (40)	148 (34)
Finland, n (%)	1129 (23)	98 (23)
Germany, n (%)	264 (5)	25 (6)
Sweden, n (%)	1601 (32)	159 (37)
Female (vs male), n (%)	2453 (50)	189 (44)
FDR (vs GP), n (%)	582 (12)	67 (16)
HLA category		
DR-DQ 3-2/4-8	1870 (38)	207 (48)
DR-DQ 4-8/4-8	965 (20)	81 (19)
DR-DQ 4-8/8-4	836 (17)	8 (16)
DR-DQ 3-2/3-2	1071 (22)	61 (14)
All others	207 (4)	13 (3)
Height Z-score; median (IQR), n	0.40 (−0.26; 1.05); 4063	0.54 (−0.10; 1.15); 380
BMI Z-score; median (IQR), n	0.20 (−0.44; 0.90); 4048	0.23 (−0.45; 0.89); 380
Height velocity Z-score; median (IQR), n	0.06 (−1.08; 1.15); 3661	0.06 (−1.09; 1.35); 343

Abbreviations: FDR, first-degree relative; GP, general population; HLA, human leukocyte antigen.

**Table 2.** Demographics for all subjects at the onset of puberty

Variable	Cohort 3, n = 4857
Site	
United States, n (%)	1924 (40)
Finland, n (%)	1081 (22)
Germany, n (%)	246 (5)
Sweden, n (%)	1606 (33)
Female (vs male), n (%)	2481 (51)
FDR (vs GP), n (%)	573 (12)
HLA category	
DR-DQ 3-2/4-8	1859 (38)
DR-DQ 4-8/4-8	947 (20)
DR-DQ 4-8/8-4	824 (17)
DR-DQ 3-2/3-2	1030 (21)
All others	197 (4)
Autoantibody status	
Negative	4397 (91)
Positive	460 (9)
Height Z-score, median (IQR); n	0.48 (−0.19; 1.11); 4602
BMI Z-score, median (IQR); n	0.26 (−0.46; 1.01); 4590
Height velocity Z-score, median (IQR); n	0.00 (−0.89; 0.89); 3595

Cohort 3 (n = 4857).

Abbreviations: FDR, first-degree relative; GP, general population; HLA, human leukocyte antigen.

### Pubertal Assessment

Pubertal onset was defined for females (breast) and males (genital development) by Tanner Stage ≥2 at age 8 and at all older ages. Tanner Stages were analyzed beginning at age 8, as they were regularly queried and documented from this age onwards. Pubertal stage was based on self-assessment performed by the child or by the parents using developmentally appropriate and validated pictures of Tanner Stages 1 to 5 [15]. In these pictures, genital development was depicted and breast development and pubic hair were depicted and described. Self-assessment was performed every 6 months during the regular TEDDY visits beginning at age 8 years until pubertal status was assessed as Stage 5 for both pubic hair/genitalia and breast development, or the child reached 15 years of age, as this is the age when individuals finish their participation in the TEDDY study. Self-assessment was done at the TEDDY clinic during the visit or at home before the visit. The form was available to be completed on paper or through the TEDDY Portal. The self-assessment was made by the parent, by the child, or the parent and child together. If there was a disagreement between the parent and child on which stage of puberty the child was in, the parent's assessment was used until the child was 10 years of age. At 10 years of age and beyond, the child's assessment was used. For a child who reported a reversal in Tanner Stage from one age to an older age, the lower Tanner Stage was replaced at the earlier age. This resulted in a nondecreasing Tanner Stage variable. Since Tanner Stage was not taken at all visits, the last reported Tanner Stage was carried forward for visits that did not have this information obtained. As of May, 2022, 94% of the females and 88% of the males reported a Tanner Stage ≥2. The median age at the onset of puberty was 11 years for girls and 11.5 years for boys. For those subjects who reported onset of puberty, the final reported Tanner Stages for females were Stage 2 (n = 267), Stage 3 (n = 510), Stage 4 (n = 1032), and Stage 5 (n = 662); the final Tanner Stages for males were Stage 2 (n = 449), Stage 3 (n = 495), Stage 4 (n = 912), Stage 5 (n = 510).

Due to TEDDY sample prioritization we were unable to measure gonadotropins, testosterone, or estrogen. Further, due to lack of universal ethics committee approval, we were unable to perform pubertal examinations on TEDDY subjects. As such, our study utilized self-examination to assess pubertal status. While self-assessment of pubertal status has been validated [15-18], we fully acknowledge the limitations of self-examination in identifying onset and progression of puberty.

### Statistical Analysis

#### Assessment of onset of puberty with either autoantibody positivity or type 1 diabetes

All statistical analyses excluded subjects with ineligible human leukocyte antigen (HLA). Measurement of autoantibodies and HLA groupings have been described previously [19]. Three different autoantibody endpoints were analyzed for Cohort 1: any persistent positive autoantibody, islet antibodies to insulin (mIAA) alone appearing as first autoantibody, and islet autoantibodies to glutamic acid decarboxylase (GADA) alone appearing as first autoantibody. If multiple antibodies became positive at the first age of autoantibody positivity or if islet autoantibodies to insulinoma antigen-2 autoantibody appeared first, these subjects only appeared as positive in the any persistent positive autoantibody endpoint per TEDDY reporting conventions. At the time of this analysis, 206 subjects in Cohort 1 developed a persistent confirmed autoantibody, 49 of these had mIAA alone as the first autoantibody, and 117 GADA alone as the first autoantibody.

Each endpoint was analyzed by a Cox proportional hazards regression model. The smoothed Tanner Stage was dichotomized and analyzed as a time-dependent variable. The age-dependent dichotomized score was either prepuberty (Tanner Stage = 1) or after onset of puberty (Tanner Stage ≥2). The primary analysis stratified the proportional hazards regression by country and included covariates for first-degree relative (yes or no), sex, and HLA. Because the age of puberty is sex dependent, analyses by sex were also done.

The progression from autoantibody-positive subjects at age 8 to type 1 diabetes was analyzed in an analogous manner to the analysis of antibodies with the age of diagnosis as the endpoint (cohort 2). There were 484 subjects in this cohort and 130 subjects have subsequently been diagnosed with type 1 diabetes.

Because of the low number of autoantibody-positive subjects in some endpoints/sex combinations, all proportional hazards analyses used the penalized maximum likelihood method called the Firth method [20] in SAS PHREG. The penalized maximum likelihood reduces the small sample bias in the estimates. A significance level of 0.01 is used to signify statistical significance in this report.

#### Assessment of pubertal growth and weight gain on autoantibody positivity or type 1 diabetes

Cohort 3 is the set of subjects who reported onset of puberty. For those subjects who were autoantibody negative at the onset of puberty, 3 different endpoints were examined in this cohort: (1) any persistent confirmed autoantibody, (2) mIAA as the first occurring autoantibody, and (3) GADA as the first occurring autoantibody. For those subjects who were autoantibody positive at the onset of puberty, type 1 diabetes was the endpoint. Time was defined as the time from the onset of puberty for these analyses.

Standardized Z-scores were computed for height, weight, body mass index (BMI), and height velocity at each visit after onset of puberty. The CDC charts were used as the reference group for height and weight. The dataset from Kelly et al [21] was used for the reference group for height velocity. Both reference groups are US-based children. All Z-scores outside of the range of (−5, 5) were excluded from analysis due to likely data reporting errors.

Cox proportional hazards analysis with the covariates indicated and changes in height and BMI Z-scores (6-month period), and height velocity (6-month period) were conducted for each of the 3 autoantibody endpoints for autoantibody negative subjects at the onset of puberty. A similar Cox analysis replacing BMI Z-score with weight Z-score was conducted using type 1 diabetes as the endpoint for autoantibody positive subjects at the onset of puberty.

#### Assessment of homeostasis model assessment of insulin resistance and speed of puberty on type 1 diabetes for multiple autoantibody and children after the onset of puberty

Some subjects became multiple antibody positive after the onset of puberty. In these subjects, the oral glucose tolerance test was recommended, and the homeostasis model assessment of insulin resistance (HOMA-IR) was calculated. Speed of puberty was assessed by time to the binary indicator of Tanner Stage ≥4 or <4; a positive HR in the analysis implies a faster rate of puberty. Cox proportional hazards analysis with the covariates age at puberty, HOMA-IR and Tanner Stage 4 (yes or no) were analyzed with type 1 diabetes as the endpoint.

#### Power calculations

Power calculations were based on the log rank test using the method of Lakatos [22]. This method is based on a Markov model that yields the asymptotic mean and variance of the log rank statistic under general conditions using a computer program described by Cantor [23]. We used the same methodology that was used for power as calculated for the prospective TEDDY cohort [13] with varying exposure proportions. For this article, we ran additional power calculations taking into consideration the long-term follow-up with varying exposure proportions.

## Results

### Assessment of Onset of Puberty and Association With Autoantibody Positivity or Type 1 Diabetes

Tables 3 and 4 show the results of the proportional hazards analysis for the 3 autoantibody endpoints for subjects who were autoantibody negative at age 8. Children reaching puberty (Tanner Stage ≥2) had lower risk (HR 0.65, 95% CI 0.45-0.93; *P* = .019) for incident autoimmunity than prepubertal children; this finding remained when males were analyzed separately (HR 0.56, 95% CI 0.34-0.93; *P* = .024). Also for the GADA first endpoint, the Tanner Stage HR was significantly less than 1 when both sexes were analyzed together (HR 0.46, 95% CI 0.28-0.76; *P* = .002) and for males separately (HR 0.41, 95% CI 0.22-0.80; *P* = .008), indicating that subjects with a Tanner Stage greater than 1 had a lower hazard for autoantibody positivity than those at Tanner Stage 1 for subjects of the same age.

**Table 3.** Proportional hazards analysis results for persistent confirmed antibodies

Number of events	Any persistent positive autoantibody		mIAA only first autoantibody		GADA only first autoantibody	
	206		49		117	
	HR estimate (95% CI)	*P*	HR estimate (95% CI)	*P*	HR estimate (95% CI)	*P*
Sex (ref = male)	0.86 (0.65; 1.14)	.291	0.99 (0.55; 1.76)	.959	0.84 (0.57; 1.23)	.363
FDR (ref = no)	2.24 (1.51; 3.34)	<.001	2.09 (0.90; 4.87)	.088	2.28 (1.36; 3.83)	.002
HLA (ref = DR4/DR3)		.141		.900		.280
DR4/DR4	0.96 (0.67; 1.38)		1.03 (0.48; 2.22)		0.82 (0.49; 1.34)	
DR4/DR8	0.80 (0.53; 1.21)		0.70 (0.28; 1.75)		0.78 (0.45; 1.37)	
DR3/DR3	0.74 (0.50; 1.08)		1.13 (0.54; 2.34)		0.81 (0.50; 1.32)	
All others	0.39 (0.16; 0.92)		0.76 (0.16; 3.58)		0.24 (0.06; 0.92)	
Tanner stage >1 (ref = no)	0.65 (0.45; 0.93)	.019	1.29 (0.61; 2.71)	.505	0.46 (0.28; 0.76)	.002

Reference cohort 1 = autoantibody negative subjects at age 8.

Abbreviations: FDR, first-degree relative; HLA, human leukocyte antigen; ref, reference variable.

**Table 4.** Proportional hazards analysis results for persistent confirmed antibodies by sex

	Any persistent positive autoantibody		mIAA only first autoantibody		GADA only first autoantibody	
	HR estimate (95% CI)	*P*	HR estimate (95% CI)	*P*	HR estimate (95% CI)	*P*
Males						
Number of events	115		24		68	
FDR (ref = no)	2.45 (1.44; 4.15)	<.001	1.75 (0.45, 6.87)	.420	2.79 (1.45, 5.34)	.002
HLA (ref = DR4/DR3)		.356		.925		.371
DR4/DR4	0.89 (0.53; 1.47)		1.67 (0.55; 5.03)		0.77 (0.39, 1.51)	
DR4/DR8	0.89 (0.52; 1.52)		1.25 (0.36; 4.39)		0.72 (0.34; 1.53)	
DR3/DR3	0.83 (0.51; 1.36)		1.44 (0.48; 4.34)		0.94 (0.52; 1.71)	
All others	0.24 (0.06; 0.95)		1.41 (0.15; 13.28)		0.07 (0.00; 1.19)	
Tanner stage >1 (ref = no)	0.56 (0.34; 0.93)	.024	1.28 (0.42; 3.92)	.667	0.41 (0.22; 0.80)	.008
Females						
Number of events	91		25		49	
FDR (ref = no)	2.14 (1.17; 3.89)	.013	2.57 (0.89; 7.44)	.81	1.80 (0.76; 4.29)	.183
HLA (ref = DR4/DR3)		.434		.744		.874
DR4/DR4	1.06 (0.63; 1.79)		0.67 (0.22; 2.08)		0.90 (0.42; 1.90)	
DR4/DR8	0.72 (0.37; 1.38)		0.42 (0.10; 1.78)		0.92 (0.41; 2.10)	
DR3/DR3	0.63 (0.34; 1.18)		0.97 (0.36; 2.61)		0.63 (0.27; 1.48)	
All others	0.61 (0.20; 1.85)		0.53 (0.07; 3.90)		0.70 (0.15; 3.26)	
Tanner stage >1 (ref = no)	0.71 (0.42; 1.22)	.216	1.18 (0.39; 3.58)	.771	0.53 (0.26; 1.08)	.080

Reference cohort 1 = autoantibody negative subjects at age 8.

Abbreviations: FDR, first-degree relative; HLA, human leukocyte antigen; ref, reference variable.

The results of the proportional hazards analysis for the type 1 diabetes endpoint for autoantibody positive subjects showed no significant effect of Tanner Stage for the diagnosis of type 1 diabetes (HR 1.20, 95% CI 0.75-1.93; *P* = .398).

### Assessment of Pubertal Growth and Weight in Relation to the Development of Autoantibodies or Type 1 Diabetes

There were 4397 subjects who were autoantibody negative at the onset of puberty. Table 5 shows the results of the proportional hazards analysis for the 3 autoantibody endpoints for subjects who were autoantibody negative at the onset of puberty. Change in BMI Z-score had a significant hazard ratio (HR 2.88, 95% CI 1.61-5.15; *P* < .001) for developing mIAA which indicates that an increase in BMI Z-score was associated with an increased hazard of developing mIAA. Analysis by gender is shown in Table 6 and suggests that the increased hazard ratio for BMI Z-score was primarily driven by female participants (HR 2.33, 95% CI 1.28-4.22; *P* = .005) and was not significant when only males were analyzed (HR 1.08, 95% CI 0.31-3.76; *P* = .902). There was no significant association between a change in height Z-score and seroconversion (Tables 5 and 6). Of 26 subjects with the finding of a persistent confirmed mIAA after puberty, 3 developed another antibody (GADA for 2 subjects approximately 12 months after seroconversion, and GADA and islet autoantibodies to insulinoma antigen-2 for 1 subject 9 and 21 months, respectively, after seroconversion). One additional subject was diagnosed with type 1 diabetes but never developed a second antibody. The median follow-up period for all 26 subjects was 25.5 months after seroconversion.

**Table 5.** Proportional hazards analysis results for persistent confirmed antibodies

Number of events	Any persistent positive autoantibody		mIAA only first autoantibody		GADA only first autoantibody	
	61		21		33	
	HR estimate (95% CI)	*P*	HR estimate (95% CI)	*P*	HR estimate (95% CI)	*P*
Sex (ref = male)	0.85 (0.50; 1.46)	.559	0.76 (0.30; 1.95)	.572	0.65 (0.32; 1.34)	.241
FDR (ref = no)	2.26 (1.10; 4.63)	.026	1.76 (0.43; 7.14)	.432	2.19 (0.82; 5.82)	.117
HLA (ref = DR4/DR3)		.460		.988		.469
DR4/DR4	0.79 (0.40; 1.55)		1.25 (0.40; 3.89)		0.56 (0.21; 1.50)	
DR4/DR8	0.58 (0.25; 1.33)		0.94 (0.24; 3.65)		0.47 (0.14; 1.54)	
DR3/DR3	0.66 (0.33; 1.31)		0.94 (0.28; 3.17)		0.69 (0.29; 1.68)	
All others	0.33 (0.06; 1.98)		1.47 (0.16; 13.57)		0.17 (0.01; 3.31)	
Age at puberty	0.82 (0.66; 1.03)	.082	0.66 (0.45; 0.98)	.038	0.83 (0.62; 1.12)	.222
Height Z-score change	9.88 (0.67; 145.7)	.096	0.43 (0.12; 1.54)	.196	0.31 (0.10; 1.00)	.050
BMI Z-score change	1.83 (1.00; 3.35)	.052	2.88 (1.61; 5.15)	<.001	0.61 (0.28; 1.33)	.211
Height velocity Z-score	0.75 (0.52; 1.09)	.130	1.14 (0.77; 1.70)	.505	1.02 (0.74; 1.41)	.906

Reference cohort = autoantibody negative subjects at the onset of puberty.

Abbreviations: FDR, first-degree relative; HLA, human leukocyte antigen; ref, reference variable.

**Table 6.** Proportional hazards analysis results for persistent confirmed autoantibodies by gender

	Any persistent autoantibody positive	
	HR estimate (95% CI)	*P*
Males		
Number of events	26	
FDR (ref = no)	1.16 (0.28; 4.79)	.836
HLA (ref = DR4/DR3)		.996
DR4/DR4	0.89 (0.31; 2.60)	
DR4/DR8	0.95 (0.29; 3.07)	
DR3/DR3	0.93 (0.34; 2.56)	
All others	0.55 (0.03; 11.29)	
Age at puberty	0.77 (0.55; 1.07)	.115
Height Z-score change	0.27 (0.06, 1.20)	.086
BMI Z-score change	1.08 (0.31, 3.76)	.902
Height velocity Z-score	1.30 (0.87, 1.94)	.209
Females		
Number of events	35	
FDR (ref = no)	3.44 (1.49, 7.93)	.004
HLA (ref = DR4/DR3)		.458
DR4/DR4	0.76 (0.32, 1.81)	
DR4/DR8	0.42 (0.13; 1.39)	
DR3/DR3	0.54 (0.20; 1.45)	
All others	0.38 (0.06; 2.45)	
Age at puberty	0.90 (0.68; 1.21)	.496
Height Z-score change	12.7 (0.69; 235.6)	.089
BMI Z-score change	2.33 (1.28; 4.22)	.005
Height velocity Z-score	0.65 (0.45; 0.95)	.026

Cohort = antibody negative subjects at the onset of puberty. FDR and HLA removed from the model for mIAA and GADA analysis.

Abbreviations: FDR, first-degree relative; HLA, human leukocyte antigen; ref, reference variable.

There were 460 subjects who were autoantibody positive at the onset of puberty. There were no significant effects for any of the growth variables on the development of type 1 diabetes in autoantibody-positive subjects (Table 7). The small number of observed cases with type 1 diabetes (n = 22) caused issues with the proportional hazards software algorithm as convergence to final estimates could not be achieved. When BMI Z-score was replaced by weight Z-score, the proportional hazards analysis did converge and there were no significant effects for any of the growth variables on the development of type 1 diabetes in autoantibody-positive subjects (Table 7). The small number of observed cases with type 1 diabetes (n = 22) limited the power to detect significant changes and the sensitivity of the convergence of the analysis suggests caution in the interpretation of this negative finding.

**Table 7.** Proportional hazards analysis results for the endpoint type 1 diabetes in the cohort of autoantibody positive subjects at the onset of puberty

Number of events	Diagnosis of type 1 diabetes	
	22	
	HR estimate (95% CI)	*P*
Gender (ref = male)	1.13 (0.42; 2.99)	.811
FDR (ref = no)	1.66 (0.49; 5.61)	.414
HLA (ref = DR4/DR3)		.221
DR4/DR4	0.45 (0.10; 1.97)	
DR4/DR8	0.48 (0.11; 2.11)	
DR3/DR3	0.67 (0.19; 2.38)	
All others	3.68 (0.71; 19.13)	
Age at puberty	1.03 (0.71; 1.47)	.891
Height Z-score change	16 (0.01; > 9000)	.508
Weight Z-score change	0.50 (0.25; 1.03)	.060
Height velocity Z-score	0.73 (0.29; 1.85)	.508

Abbreviations: FDR, first-degree relative; HLA, human leukocyte antigen; ref, reference variable.

#### Assessment of HOMA-IR and speed of puberty in multiple autoantibody–positive children

After the onset of puberty, 245 subjects were persistently positive for multiple islet autoantibodies. Of these 245 subjects, 17 were subsequently diagnosed with type 1 diabetes. There was no significant effect for either Tanner Stage 4 or HOMA in this analysis (Table 8). Because of the low number of events, analyses by sex were not conducted. The small number of observed cases with type 1 diabetes limited the power to detect significant changes.

**Table 8.** Proportional hazards analysis results for type 1 diabetes in the cohort of subjects with multiple persistent autoantibodies at the onset of puberty

Number of events	Diagnosis of type 1 diabetes	
	17	
	HR estimate (95% CI)	*P*
Gender (ref = male)	1.34 (0.43; 4.11)	.615
FDR (ref = no)	1.46 (0.49; 0.26; 8.21)	.669
HLA (ref = DR4/DR3)		.521
DR4/DR4	0.14 (0.01; 3.07)	
DR4/DR8	0.95 (0.17; 5.37)	
DR3/DR3	1.13 (0.27; 4.66)	
All others	3.39 (0.42; 27.3)	
Age at puberty	1.17 (0.74; 1.83)	.501
Tanner stage ≥4 (ref = no)	0.36 (0.04; 3.10)	.353
HOMA-IR	0.84 (0.46;1.54)	.578

Abbreviations: FDR, first-degree relative; HLA, human leukocyte antigen; ref, reference variable.

## Discussion

The analyses we conducted showed that an increase in BMI Z-score is associated with a significantly increased risk of developing islet autoimmunity. In particular, this was seen for mIAA, and not for all autoantibodies assessed. Our results show that weight gain during puberty may favor the development of autoimmunity in a population with increased genetic risk for type 1 diabetes. It was surprising that an increase in BMI Z-score was associated with the development of mIAA, an autoantibody which is usually seen at an earlier age, and we can only speculate about possible reasons for this. It is possible that this autoantibody plays an important role not only in early childhood, but also in adolescents who gain weight, and could be triggered by weight gain/insulin resistance. To the best of our knowledge, this observation has never been made before and it would be interesting to investigate this in further collectives. Increased weight is often associated with an increase in growth velocity. However, there was no significant relationship between the height prior to seroconversion and weight gain (data not shown). The finding regarding the change in BMI Z-score is consistent with weight findings in the first 12 months of life [24]. For the other factors we investigated, there was no significant association with the development of autoimmunity or type 1 diabetes. While there was no significant association the low number of events limited the statistical power and should not be interpreted as a lack of effect. In particular, there was no association between the age at the onset of puberty and autoimmunity or type 1 diabetes. We also could not identify change in weight, height, or height velocity during puberty as risk factors in the progression of autoimmunity to clinically manifest type 1 diabetes although the number of cases with type 1 diabetes was low (n = 22). In this analysis we could not confirm an increased risk of seroconversion to islet autoimmunity or progression to clinical type 1 diabetes related to onset of puberty. In another large cohort study of children followed prospectively in the Finnish type 1 Diabetes Prediction and Prevention study (DIPP), puberty was associated with an increased rate of progression from islet autoimmunity to type 1 diabetes but not with the incidence of islet autoimmunity [25]. In the DIPP study, timing of puberty was based on SITAR-modeled growth data [26]. Since the TEDDY cohort is restricted to children with increased genetic risk, we cannot exclude that this may be different in children with other genetic composition. Previous studies have reported that children with lower genetic risk may have a later onset of type 1 diabetes than high-risk children, and also a higher weight at onset [27].

TEDDY children are followed closely from islet autoantibody positivity to onset of type 1 diabetes, and are diagnosed at an early stage of disease, often without symptoms [28, 29]. This could also influence the possibility to detect an impact of puberty and insulin resistance on progression to clinical type 1 diabetes.

### Limitations and Strengths

The TEDDY study is the only large international study that prospectively follows children at increased genetic risk for type 1 diabetes from birth and collects longitudinal data on growth, weight development and pubertal development in an at-risk cohort, which made the longitudinal analyses of the association of puberty with development of islet autoimmunity and type 1 diabetes possible. From the ninth birthday onwards, information about Tanner Stage was consistently available for about 95% of the subjects, such that missing data were unlikely to bias the analysis. However, as noted from the outset, a major limiting factor was that data on pubertal development were based only on self-assessment. Given the unique regulatory and ethical constraints in several of the participating TEDDY countries, an examination of the Tanner Stages by study personnel was considered unethical, and self-assessment was the only possible solution across the entire collaborative. In addition, laboratory measurement of gonadotropins and/or estradiol/testosterone levels was proposed but was ultimately not pursued due to funding limitations and the relatively high sample volumes that would have been required. While self-assessment is a validated tool, we acknowledge that other modalities would have been superior for determining the exact stages of puberty, pubertal progression, and the speed of puberty. According to a large Danish analysis, girls and their parents tend to underestimate, whereas boys overestimate their pubertal stage [15]. Notably, boys in our cohort reported onset of puberty at ages consistent with prior studies of pubertal onset, while girls reported onset of thelarche nearly 1 year later than what is typically observed [26].

Another limitation was the low number of children progressing to diabetes during this period, as most of the study cohort had progressed to type 1 diabetes earlier in the study. The small number of children who developed diabetes during or shortly after the onset of puberty markedly reduced the statistical power of several analyses and resulted in several nonsignificant findings that should be interpreted with caution.

## Conclusions

Weight gain during puberty, primarily in girls, may favor the development of islet autoimmunity in children and adolescents at increased genetic risk for type 1 diabetes. Self-assessment of the onset of puberty and rate of pubertal progression were not associated with autoimmunity or the progression to type 1 diabetes in this cohort of children with increased genetic risk. Additional studies are needed to fully explicate the associations between puberty and islet autoimmunity and to improve our understanding of the mechanisms involved. Assessment of the Tanner Stages by medical staff and additional laboratory investigation, such as gonadotropins, sex hormones, and parameters investigating insulin resistance and β-cell function (eg, area under the curve of the first-phase insulin secretion, glucose disposition index, and the HOMA-β) should be performed as part of future longitudinal cohort studies.

## Data Availability

“Data from The Environmental Determinants of Diabetes in the Young (https://doi.org/10.58020/y3jk-x087) reported here will be made available for request at the NIDDK Central Repository (NIDDK-CR) website, Resources for Research (R4R), https://repository.niddk.nih.gov/.”

## Clinical Trial Information

The TEDDY study is registered as of January 17, 2006, under the ClinicalTrials.gov ID NCT00279318.

## Abbreviations

BMI: body mass index
GADA: glutamic acid decarboxylase
HLA: human leukocyte antigen
HOMA-IR: homeostasis model assessment of insulin resistance
mIAA: islet antibodies to insulin
TEDDY: The Environmental Determinants of Diabetes in the Young
